# Combine and Conquer: With Type 2 Diabetes Polypharmacy Is Essential Not Only to Achieve Glycemic Control but Also to Treat the Comorbidities and Stabilize or Slow the Advancement of Diabetic Nephropathy

**DOI:** 10.1155/2022/7787732

**Published:** 2022-08-04

**Authors:** David S. H. Bell

**Affiliations:** Southside Endocrinology, 1900 Crestwood Blvd, Suite 201, Irondale, AL 35210, USA

## Abstract

The concept of polypharmacy in the type 2 diabetic patient is both historic and redundant. A combination of three or more medications usually at doses which are less than those utilized for monotherapy is efficacious not only in the therapy of hyperglycemia but also in the therapy of the comorbidities of hypertension and hyperlipidemia. In addition, multiple medications are now accepted as being necessary to reduce albuminuria and decelerate the decline in renal function in the patient with diabetic nephropathy.

## 1. Introduction

Historically, the use of polypharmacy (more than five different medications per patient) was not encouraged in either the diabetic or the nondiabetic patient [1]. The reasons that were given for this were potential mistakes in the prescribing or administration of drugs, drug-drug interactions, and drug-disease interactions [2]. However, in the therapy of diabetes, its comorbidities, and its complications, the use of multiple medications, often in lower doses than are recommended for monotherapy, is widely accepted because of proven efficacy and reductions in the side effects of therapy due to lower dosing [3].

## 2. Combined Oral Therapy in the Therapy of Type 2 Diabetes

In 2006, I laid out the case for combination oral therapy as first-line therapy for the type 2 diabetic patient [4]. I suggested that to achieve maximal lowering of the HbA1c without the occurrence of hypoglycemia, the combination of metformin and a thiazolidinedione should be utilized [4]. Prior to this, due to the lack of evidence of longevity of the effects of sulfonylureas and metformin and the unavailability of other efficacious drugs, combination therapy was discouraged [5–7]. Later, our group was the first to describe the efficacy of triple oral therapy (metformin, a thiazolidinedione, and a sulfonylurea) which did not only achieve better glycemic control but also slowed progress toward the initiation of insulin therapy in the type 2 diabetic subject [8]. On follow-up of these patients, we found that after 37 months, 35 patients were still well controlled (HbA1c 6.9 ± 0.3%) and only 9 were uncontrolled (HbA1c 8.8 ± 0.5%) and needed to advance to insulin therapy [8]. We later found that the reason for the prolonged improvement in glycemic control was the ability of the thiazolidinedione to improve beta cell function [9]. Our finding of thiazolidinedione induced improvement in beta cell function which was later proven in a prospective study of subjects who had failed the combination of metformin and a sulfonylurea and who were randomized either to the addition of the thiazolidinedione rosiglitazone or to a single once daily injection of premixed insulin. In this study where changes in the HbA1c were equal, the thiazolidinedione returned first phase insulin release which did not occur with the addition of insulin therapy [9]. In addition, the disposition index and the proinsulin-to-insulin ratio improved confirming an overall improvement in beta cell function [9].

**Figure 1:**
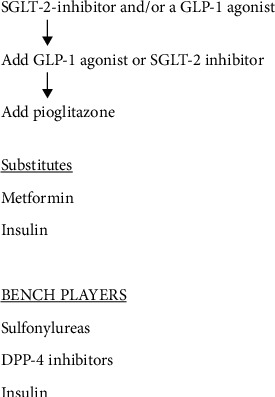
Algorithm for glucose control in the type 2 patient.

Since then, there have been multiple studies of triple oral therapy in type 2 diabetic subjects with almost all of these studies utilizing metformin in combination with sulfonylureas, DPP-4 inhibitors, thiazolidinediones, and SGLT-2-inhibitors and more recently injections of various GLP-1 receptor agonists [10]. Furthermore, oral semaglutide has been shown to be efficacious when added to both metformin and an SGLT-2 inhibitor [11, 12]. In addition, due to the tachyphylaxis that occurs with both metformin and sulfonylureas, it has been suggested that the ideal initial therapy in the type 2 diabetic patient should be a combination of metformin, pioglitazone, and the injectable GLP-1 receptor agonist exenatide [6, 8, 13]. This combination not only produces a greater reduction of HbA1c but also a more durable HbA1c reduction [14]. Therefore, triple therapy used either initially or later in the course of type 2 diabetes has been shown to be efficacious and should probably be utilized as early as possible in the therapy of the type 2 diabetic patient (Figure 1). In addition, with newer drugs, there are nondiabetic advantages, e.g., metformin in the therapy of cancer, pioglitazone in the therapy of fatty liver disease, and SGLT-2s in the therapy of heart failure [15–17].

## 3. Combination Oral Therapy for Hypertension in the Type 2 Diabetic Patient

Over 75% of type 2 diabetic patients also have hypertension due to the high insulin levels associated with insulin resistance increasing salt and water retention and the association of insulin resistance with increased sympathetic nervous system activity [18].

Multiple antihypertensives used in combination to achieve target blood pressure levels have been widely utilized for many years in the type 2 diabetic patient. Ideally, the drugs utilized should be from different classes and include a diuretic [19]. The usual combination is a thiazide diuretic, a long-acting calcium channel blocker and a blocker of the renin-angiotensin system. When patients remain hypertensive on a triple-therapy regimen, they are classified as having “resistant hypertension,” and “resistant hypertension” is more common in diabetic subjects [20].

With resistant hypertension, aldosterone excess has been shown to be common, and the use of a steroidal mineralocorticoid receptor antagonist, such as spironolactone or eplerenone, has been shown to be efficacious in the majority of cases [21]. Since in a type 2 diabetic patient hyperkalemia is more likely to occur with a steroidal mineralocorticoid receptor antagonist such as spironolactone or eplerenone, caution needs to be utilized through regular monitoring of serum potassium levels. In the future with the availability of the nonsteroidal selective mineralocorticoid receptor agonist, finerenone, which is associated with less hyperkalemia than the steroidal mineralocorticoid receptor antagonists, may be more safely prescribed in the therapy of resistant hypertension especially in the diabetic patient [22].

The choice of the calcium channel blocker is also important. The dihydropyridine calcium channel blockers (CCBs) tend to stimulate the sympathetic nervous system more than the nondihydropyridine CCBs so that lower blood pressure levels can usually be obtained with less side effects utilizing the nondihydropyridine CCBs [23]. In addition, with the dihydropyridine CCBs, albuminuria may increase, whereas urine albumin levels are stable and may even decrease with the nondihydropyridine CCBs [24]. In addition, with the nondihydropyridine CCB verapamil, it has been shown that, in both type 1 and type 2 diabetes, by decreasing the expression of thioredoxin-interacting protein, beta cell apoptosis is decreased and beta cell survival is improved [25, 26]. Therefore, in the presence of diabetes, the preferred CCB is the nondihydropyridine CCB verapamil.

If a beta blocker is utilized in the diabetic subject, a vasodilating beta block such as carvedilol or nebivolol which vasodilate through stimulation of the B_3_-receptor should be utilized since through vasoconstriction nonvasodilating beta blockers increase insulin resistance, decrease insulin release, worsen glycemic control, and accelerate progression to microalbuminuria [27, 28].

**Figure 2:**
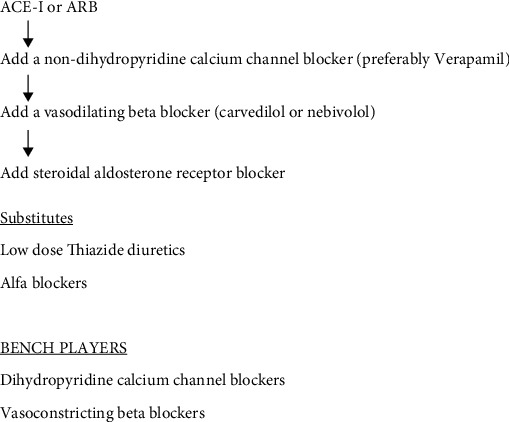
Algorithm for hypertension treatment in the type 2 diabetic patient.

Also, in the diabetic subject, thiazide diuretics even at lower doses also increase insulin resistance and decrease insulin release [29]. This is particularly true when a thiazide diuretic is utilized in combination with a vasoconstricting beta blocker where the effect on hyperglycemia is additive [30]. The use of a small dose of a steroidal aldosterone receptor blocker rather than a thiazide diuretic to treat hypertension in the diabetic patient is preferable since it is not associated with drug-induced hyperglycemia [31] (Figure 2).

## 4. Combination Oral Therapy for Therapy of the Dyslipidemia Associated with Type 2 Diabetes

The most important component of the therapy for the dyslipidemia associated with type 2 diabetes is lowering the low-density lipoprotein (LDL) cholesterol level. American Association of Clinical Endocrinology (AACE) guidelines recommend lowering of LDL to 55 mg/dl or below in diabetic patients with established coronary artery disease [32]. The European guidelines go even further recommending that if the LDL is lowered to 55 mg/dl or below and after two years cardiac events continue to occur, the goal for LDL should be 35 mg/dl [33].

A starting dose of a statin usually lowers the LDL cholesterol level by as much as 50% [34]. However, even with a powerful statin, the goal of an LDL of 55 mg/dl is rarely achieved with monotherapy. In fact, doubling the dose of a statin will only lower the LDL by another 6% and will be associated with a higher incidence of side effects [35]. Therefore, the goal of 55 mg/dl or lower for LDL is often unobtainable with statin monotherapy and other lipid-lowering drugs utilized in combination with a statin are required.

The addition of ezetimibe will lower the LDL by at least another 10% [36, 37]. If a further reduction in LDL is needed, bempedoic acid will further lower the LDL by as much as an additional 15% when added to maximally tolerated statin therapy [38]. In many patients who cannot tolerate a statin, even at a lower dose, the use of the combination of ezetimibe and bempedoic acid lowering of the LDL to target may be achieved [39].

Therefore, with the availability of ezetimibe and bempedoic acid, LDL targets can in most situations be achieved with triple oral therapy (statin/ezetimibe/bempedoic acid). Should the LDL not be at goal, then the addition of a PCSK9-inhibitor should be considered [40].

If further LDL lowering or replacement of drugs that cannot be tolerated is needed, the addition of colesevelam which lowers LDL by 16% and C-reactive protein by 22% and lowers the HbA1c by 0.26% can be used. While colesevelam is a bile acid sequestrant, it has been modified to limit gastrointestinal side effects which results in greater patient compliance [41].

The addition of a fish oil derivative icosapent ethyl which not only lowers triglycerides but independent of triglyceride levels lowers both cardiac events and cardiac mortality, especially in the diabetic patient, may be needed and should be added if triglyceride levels are over 150 mg/dl [42].

**Figure 3:**
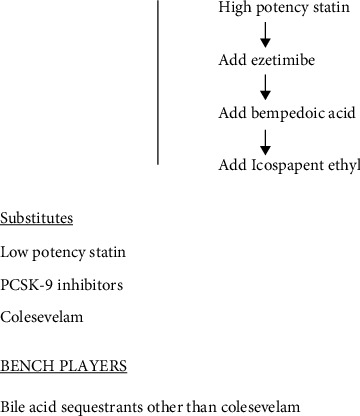
Algorithm for treatment of hyperlipidemia in the type 2 diabetic patient.

Thus, to satisfactorily control hyperlipidemia and especially LDL cholesterol in the diabetic patient as many as four or more, oral agents may be required (Figure 3).

## 5. Diabetic Nephropathy

Blockers of the renin-angiotensin-aldosterone system (RAAS) are efficacious in both decreasing proteinuria and decelerating the decline in renal function in both the type 1 and type 2 diabetic patients who have diabetic nephropathy. Glomerular pressure is decreased with ACE inhibitors through dilatation of the efferent arteriole which by decreasing the glomerular pressure results in decreases in proteinuria as well as deceleration of the progression to renal failure through a decrease in podocyte injury and deceleration of the glomerulosclerosis [43].

At present, only the ACE inhibitor captopril is approved for the therapy of nephropathy associated with type 1 diabetes and the angiotensin II receptor blocker (ARB) losartan for the therapy of nephropathy associated with type 2 diabetes [44, 45]. However, renin-angiotensin-aldosterone system (RAAS) blockers used as monotherapy, with the possible exception of the therapy of early microalbuminuria, are not powerful enough as monotherapy to eliminate proteinuria and/or to decelerate the decline in renal function and additional therapy is needed.

With or without RAAS blockers, proteinuria decreases and the decline in renal dysfunction decelerates with SGLT-2 inhibitors. Through blockade of the SGLT-2 receptor in the proximal tubule, SGLT-2 inhibitors block not only the reabsorption of glucose but also the reabsorption of sodium which increases the quantity of sodium presenting to the macula densa which is situated in the juxtaglomerular apparatus at the top of the thick ascending limb of the loop of Henle at the point where the loop of Henle meets the distal convoluted tubule. This increase in urinary sodium content is misinterpreted by the macular densa to be due to an increase in plasma volume. This misinterpretation results in not only a decrease in RAAS activity from the renin secreting cells in the juxtaglomerular apparatus but also a negative “tubuloglomerular” feedback. Anatomically, the juxtaglomerular apparatus is in close proximity to the afferent glomerular arteriole which results in constriction of this arteriole which decreases both intraglomerular pressure and hyperfiltration [46]. The vasoconstriction of the afferent arteriole complements the decrease in glomerular pressure achieved by RAAS blocker-induced dilation of the efferent arteriole. Other possible etiologies for renoprotection with SGLT-2 inhibitors are that due to decreased glomerular pressure and glomerular wall tension, there are decreases in inflammation, oxidative stress, and fibrosis [46, 47].

Another mechanism for the renoprotective effect is that SGLT-2 inhibitors increase the production of ketone bodies which are preferentially utilized by the kidney as a source of energy. The shift away from the use of free fatty acids and glucose as a source of energy toward the greater use of ketone bodies results in a decrease in the workload of the kidney which in turn results in a deceleration of the decline in renal function [48].

The SGLT-2 receptor blockers that have been studied in combination with RAS blockade to asses renoprotection are canagliflozin, empagliflozin, and dapagliflozin. In a double-blind, placebo-controlled study, type 2 diabetic subjects with albuminuria and chronic kidney disease and who were already utilizing RAAS blockers were randomized to either placebo or canagliflozin 100 mg daily. This study was prematurely terminated due to a positive interim analysis where the composite of dialysis, transplantation, developing a GFR of under 15 ml/min, doubling of the serum creatinine, or death (cardiac or renal cause) was significantly reduced by 30% (95% CI 0.59-0.82, *p* = 0.001). In addition, stroke, cardiovascular, death, and myocardial infarction which are increased with renal dysfunction were reduced by 20% (95% CI 0.67-0.95, *p* = 0.001) and hospitalization for heart failure by 39% (95% CI 0.47-0.80, *p* = 0.001) [49].

Empagliflozin was utilized in the EMPA-REG outcome trial where 525 subjects in the empagliflozin group who were randomized to 10 mg or 25 mg of empagliflozin were compared to 388 subjects randomized to placebo. In this study, doubling of the serum creatinine was reduced by 44% and initiation of dialysis or transplantation by 55% [50].

The DAPA-CKP trial enrolled subjects who had an albumin-to-creatinine ratio of 200 mg-500 mg/g and an estimated GFR of 25-75 ml/min and of whom 68% had diabetes. These subjects were randomized either to dapagliflozin 10 mg once daily or placebo. The end-point was a composite of a 58% decline in GFR, development of end-stage renal disease (dialysis or death), or renal or cardiac death. This end-point over an average of 2.4 years was reduced by 36% (95% CI 0.52-0.79) in those with diabetes and 50% in those without diabetes group (95% CI 0.53-0.92) [51].

Mineralocorticoid receptor antagonists (MRAs) reduce albuminuria and preserve renal function. The mineralocorticoid receptor is a nuclear receptor expressed in many tissues including the kidney, immune cells, and fibroblasts [52]. In the kidney, the MR-receptor plays a major role in fluid, electrolyte, and hemodynamic homeostasis in addition to tissue remodeling. Overactivity of the MR-receptor leads to inflammation and fibrosis, and deceleration of the MR-receptor activity leads to decreased albuminuria even when used in combination with RAAS blockers [53, 54]. However, the steroidal mineralocorticoid receptor antagonists cause hyperkalemia especially in diabetic patients and in those with decreased cardiac or renal function particularly when used in combination with RAAS blockers (RR 2.17, 95% CI 1.47-3.22) [55]. However, the availability of the nonsteroidal mineralocorticoid-receptor antagonist finerenone has shown an efficacy that is equal to that of the steroidal mineralocorticoid receptor antagonists but with a lower rate of hyperkalemia even when used in combination with RAAS blockade [56].

Thus, in the patient with diabetic nephropathy when RAAS blockade fails to return the urine albumin level to normal, the addition of a SGLT-2 antagonist or a mineralocorticoid receptor blocker can be added. If the urine albumin level is still elevated, then advancement to triple oral therapy with RAAS blocker MRA agonists and SGLT-2 receptor blockers should be considered. While to date there is no hard evidence that triple therapy would be efficacious in the therapy of diabetic nephropathy, there is a retrospective report of a prospective study of patients with diabetic nephropathy already on a RAS inhibitor treated with finerenone where there was a 19% decrease (95% CI 0.72-0.92) in the frequency of a decline in the glomerular filtration rate of over 40%. In this study, a small number of subjects were also taking SGLT-2 inhibitors, and these subjects were more likely to have a greater decrease in albuminuria (41% versus 32%, *p* = 0.04) [57].

**Figure 4:**
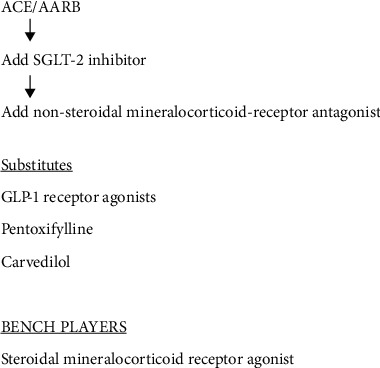
Algorithm for therapy of diabetic nephropathy.

Therefore, the combination of RAAS inhibitors, SGLT-2 inhibitors, and MR-receptor blockers is likely to be utilized in diabetic patients who do not reduce urine albumin levels to below 30 mg/g creatinine since below this level advancement to renal failure is very unlikely and cardiovascular events are decreased [58, 59] (Figure 4).

GLP-1 receptor agonists have also been shown to have a renoprotective effect. While GLP-1 receptors have been demonstrated in the kidney, the receptors in humans are confined to the proximal tubule and preglomerular vascular smooth muscle cells [60]. The direct renoprotective effects of GLP-1 receptor agonists are thought to be due to natriuresis through stimulation of the proximal tubule, modulation of AMP-PKA signaling, inhibition of the renin-angiotensin system, decreased renal hypoxia, decreased glomerular atherosclerosis, endothelial dependent vasodilatation, and tubuloglomerular feedback [61]. As a result of these effects in a meta-analysis of 56,004 patients, macroalbuminuria was reduced by 24% (95% CI 0.68-0.86, *p* = 0.003) with GLP-1 receptor agonists [62]. Semaglutide in a prospective, blinded, placebo-controlled study of 3,297 patients showed that compared with placebo, there was a 36% reduction (95% CI 0.46-0.88, *p* = 0.005) in new or worsening nephropathy in spite of 76% (95% CI 1.11-2.78) increase in the microvascular complication of retinopathy [63].

Another therapy that is a candidate to reduce albuminuria and frequency of renal decompensation is pentoxifylline [64]. This older drug through TNF*α* suppression has been shown to decrease macroalbuminuria, but not microalbuminuria, and decelerate the progression to renal failure in patients with diabetic nephropathy.

Another candidate for renoprotection is the vasodilating beta blocker carvedilol which has been shown to reduce albuminuria. In the GEMINI study of hypertensive type 2 diabetic subjects, carvedilol compared with placebo reduced albuminuria by 16.2% (95% CI -25.3, -5.9, *p* = 0.003) [65]. This decline in albuminuria was initially thought to be due to antioxidant activity but was later theorized to be due to beta-3 adrenergic receptor blockade inducing vasodilatation of the efferent artery of the glomerulus.

Therefore, as many as six currently available drugs could be utilized to decrease proteinuria and decelerate the decline in renal function in the patient with diabetic nephropathy. At the present time, the most effective combination for diabetic nephropathy appears to be that of a RAAS-inhibitor, a nonsteroidal mineralocorticoid receptor antagonist, and an SGLT-2 inhibitor. However, second-line drugs such as GLP-1 receptor agonists, pentoxifylline, and carvedilol could be used either as a replacement for or as an addition to these medications (Figure 4).

## 6. Conclusion

The concept of polypharmacy is redundant as far as therapy for type 2 diabetes, its comorbidities, and its complications are concerned. The use of multiple drug combinations in randomized placebo-controlled trials has been shown to be efficacious in treating not only hyperglycemia but also the comorbidities of hypertension and hyperlipidemia in the type 2 diabetic patient as well as the complication of diabetic nephropathy. In spite of this well-documented evidence of decreased morbidity and mortality with these medications, patients with advanced diabetes despite having the worst renal and cardiovascular prognosis are at high risk of being undertreated, and this is universal and not associated with type of clinical setting in which they are treated [66].

## Data Availability

The data that support the findings of this study are available from the corresponding author upon reasonable request.
